# The British Medical Association and Its Critics

**Published:** 1913-10-04

**Authors:** 


### The Hospital

The Workers' Newspaper of Administrative Medicine and Institutional Life, Administration, National Insurance and Health.

No. 1423, Vol. LV. SATURDAY, OCTOBER 4, 1913. PRICE ONE PENNY.

Z30051

### THE BRITISH MEDICAL ASSOCIATION AND ITS CRITICS.

When the dispute between the medical profession and the Chancellor of the Exchequer on the subject of panel practice ended early in this present year with the defeat of the former, the demonstration of the plasticity and impotence in the Chancellor's hands of the British Medical Association was so complete as thoroughly to convince many of its members that the Association is useless for upholding the interests of the medical profession. For the moment we are not concerned with the question whether this dissatisfaction is well founded or with the question whether any alternative corporate society could do better; we merely note that deep-rooted discontent exists and has been simmering ever since January last. Matters have come to a head in the Wandsworth division of the British Medical Association, which has passed by twenty-one votes to fourteen at a special meeting a resolution which is practically a vote of disapproval of the policy of the Association. The logical step for these dissatisfied practitioners to adopt is to cut themselves loose from the Association by resigning their membership, and this we gather a good many of them are doing. The Wandsworth doctors have, in fact, set an example to those who still adhere to the views which the British Medical Association abandoned in such a hurry nine months ago; and numerous resignations are reported from several centres as a result of the Wandsworth resolution.

The situation thus disclosed is at present probably not very serious for the Association as far as the actual number of accomplished defections is concerned But the Association, after the crises which it has passed through during the last two years, cannot possibly regard with equanimity any reduction in its numbers or any deep cleavage within its ranks. It is not surprising, therefore, that it was thought worth while to send Dr. Cox, the medical secretary, to attend the special meeting and to attempt to persuade the members not to pass the resolution submitted to them. As it turned out, Dr. Cox's oratory proved unavailing, and a somewhat heated discussion has gone on since between himself and Dr. Edwin Smith, the mover of the resolution, as to the treatment which he received from the meeting.

Dr. Cox's position is exceedingly awkward and deserving of sympathy, for he found himself practically compelled and in duty bound to go to Wandsworth to uphold the very thing (panel practice on Mr. Lloyd George's terms) which the Association a year ago was officially declaring to be "derogatory to the profession and against the public interest." It is also his business to try to retain within the Association both panel and non-panel practitioners and to serve the interests of both. The impossible position in which he thus finds himself is not his fault, but that of the majority of the members, who talked big but ran away when the time came for acting.

Whether or not Dr. Cox was treated with courtesy and consideration at the Wandsworth meeting is, of course, a matter of opinion which only those who were present are qualified to decide. He complains of interruptions and of being cut short by the Chairman; the reply is that he exceeded the time limit set for speeches and declined to answer questions which members wished to ask him. Another somewhat barren controversy is waged about the number of members present. It is easy enough to say that an attendance of forty members out of 250 is a poor meeting; so it would be for miners on strike, but for a British Medical Association branch meeting it is an uncommonly good muster.

At all events, it is clear that the question of the panel threatens very gravely the future of the Association. The National Medical Guild, as our own columns bear witness, and other organisations, unheard of until the Insurance Act came into being, are attracting many members who have either lost confidence in or have actually resigned from the Association. Dr. Edwin Smith, the mover of the Wandsworth resolution, and his numerous sympathisers in the profession have taken a bold step, and the result of his energy and initiative remains to be seen. At least it shows that a considerable

W1
NO69X

section of the profession, containing men of high principles and exceptional professional ability, is profoundly dissatisfied with the working of the Insurance Act in so far as medical benefit is concerned. It shows that this feeling is coming to a head, at least in London, and probably in some other large towns, and that the folly of coercing a self-sacrificing profession like that of medicine by the bluff and chicanery of last January may yet be brought home to the principal agents in that campaign. After the exhibition of disunion and moral cowardice which the bulk of the practitioners then gave, it is hard to believe that they will ever now stiffen their backs sufficiently to achieve any purpose requiring courage, endurance, and mutual assistance. But it is something to know that there is a minority for whom a solemn pledge does mean something sacred, whose principles are not liable to changes at a moment's notice. If it turns out that for these men there is no hope and no room in the British Medical Association so much the worse for the Association.

